# Silicon mitigates K deficiency in maize by modifying C, N, and P stoichiometry and nutritional efficiency

**DOI:** 10.1038/s41598-023-44301-5

**Published:** 2023-10-07

**Authors:** Milton Garcia Costa, Renato de Mello Prado, Marcilene Machado dos Santos Sarah, Antônia Erica Santos de Souza, Jonas Pereira de Souza Júnior

**Affiliations:** School of Agricultural and Veterinarian Sciences, São Paulo State University (Unesp), Via de Acesso Prof. Paulo Donato Castellane S/N, Jaboticabal, 14884-900 Brazil

**Keywords:** Plant development, Plant physiology

## Abstract

Potassium (K) deficiency in maize plants damages the nutritional functions of K. However, few studies have investigated the influence of K on C:N:P stoichiometry, the nutritional efficiency of these nutrients, and whether the mitigating effect of Si in plants under stress could act on these nutritional mechanisms involved with C, N, and P to mitigate K deficiency. Therefore, this study aimed to evaluate the impact of K deficiency in the absence and presence of Si on N and P uptake, C:N:P stoichiometric homeostasis, nutritional efficiency, photosynthetic rate, and dry matter production of maize plants. The experiment was conducted under controlled conditions using a 2 × 2 factorial scheme comprising two K concentrations: potassium deficiency (7.82 mg L^−1^) and potassium sufficiency (234.59 mg L^−1^). These concentrations were combined with the absence (0.0 mg L^−1^) and presence of Si (56.17 mg L^−1^), arranged in randomized blocks with five replicates. Potassium deficiency decreased stoichiometric ratios (C:N and C:P) and the plant’s C, N, and P accumulation. Furthermore, it decreased the use efficiency of these nutrients, net photosynthesis, and biomass of maize plants. The results showed that Si supply stood out in K-deficient maize plants by increasing the C, N, and P accumulation. Moreover, it decreased stoichiometric ratios (C:N, C:P, N:P, C:Si, N:Si, and P:Si) and increased the efficiencies of uptake, translocation, and use of nutrients, net photosynthesis, and dry matter production of maize plants. Therefore, the low nutritional efficiency of C, N, and P caused by K deficiency in maize plants can be alleviated with the supply of 56.17 mg L^−1^ of Si in the nutrient solution. It changes C:N:P stoichiometry and favors the use efficiency of these nutrients, which enhances the photosynthesis and sustainability of maize.

## Introduction

The acceleration in the world population growth is responsible for an exponential increase of approximately 35–56% in food demand^1^. At the same time, climate change has reduced crop yield and food supply worldwide through droughts or excessive rainfall^2^.

Excessive rainfall can damage soil nutrient balance, resulting in the loss of cations of nutrients such as potassium by leaching^3^. In addition, drought can decrease K diffusion in the soil and the ion-root contact, consequently decreasing nutrient uptake by the plant^4,5^. Moreover, a large amount of K is usually removed from the soil when the whole plant is harvested, and the frequent use of low doses of potassium fertilizers increases K deficiency^6^.

Thus, it is evident that K deficiency in agricultural production systems is worsening over time and may affect different known functions of K in the plant, especially in enzyme activity, carbohydrate transport, and water use efficiency^7^. Even though these damages caused by K deficiency are well known, the lack of this nutrient affects other nutrients, such as N and P. It decreases their uptake^8–12^ and use efficiency^9^, aggravating the biochemical disturbances involved in the photosynthetic process and the translocation of assimilates^13^ further hampering crop growth and production^13–16^.

This decrease in plant growth may be caused by the loss of homeostasis of nutrients essential to the plant (C, N, and P) due to the stress caused by K deficiency. However, it has yet to be studied, especially in Poaceae such as maize. Moreover, it still needs to be proven. This suspicion emerged after a recent study in cotton plants showed that the stress promoted by K deficiency modified the C:N ratio in the plant^17^. A similar fact was also reported for other plant stresses, such as saline^18^ and water stress^19–24^ which also modified stoichiometric ratios involving C:N:P. These stresses caused losses in nutritional homeostasis, consequently decreasing these plants’ nutritional efficiency and growth. Thus, it is opportune to better understand whether the weight of K deficiency in the drastic reduction in the growth of maize plants is also caused by the nutritional component involving the homeostasis of N, P, and C.

Furthermore, a recent study revealed that K deficiency changes the root system, reducing the development of root hairs^25^ and consequently decreasing the uptake of other nutrients by plants. In addition, a study conducted with different K doses demonstrated that an increase in its dosage led to an increase in other nutrients^26^. Thus, it indicates that K deficiency hampers the uptake of other nutrients and consequently disrupts the nutritional balance. Overall, the precise mechanisms by which K deficiency induces C:N:P imbalance in maize plants may involve a combination of impaired nutrient uptake, altered metabolic processes, and compromised root function.

The biological damage of K deficiency in maize plants must be minimized sustainably without risking the environment. Therefore, there is the option of using Si. Previous studies have reported that Si can alleviate K deficiency in some species, such as forage^27^, sorghum^28^, barley^29^, soybean^30^, peanut^31^ beans^32,33^ and maize^34^. Moreover, it increases K use efficiency in peanut^31^, basil^35^, bean^32^ and maize^34^. However, it was explained exclusively by the benefits of Si in alleviating oxidative stress, while impacts on C:N:P stoichiometry were not addressed. Thus, there is a question regarding whether or not the growth improvement promoted by Si in these K-deficient maize has a nutritional component involving C:N:P homeostasis.

Given the above, it is important to better understand the underlying effects of the impact of K deficiency (and its relationship with Si) on the nutrition of other structural nutrients, such as C, N, and P, which are extremely important for plant metabolism. Therefore, it is worth testing the following hypotheses: (i) K deficiency promotes a decrease in the uptake of N and P, which was observed in plants such as rapeseed^6^. These may be important nutrients in the photosynthetic process, modifying C:N:P stoichiometry and aggravating the plant’s ability to efficiently use these nutrients, consequently decreasing plant biomass production. If this is confirmed, (ii) the use of Si can reverse these processes by increasing N and P uptake while also favoring C:N:P homeostasis, optimizing photosynthesis and resulting in a highly efficient use of these nutrients. Thus, it increases dry matter accumulation, especially in the plant shoot. However, it is believed that these Si benefits do not occur in plants with enough K. Hence, there is also the hypothesis that (iii) the Si benefits predominate in plants under stress compared to those without stress since Si is not considered a plant nutrient^36^.

In order to test these hypotheses, this study aimed to evaluate the K deficiency impact (in the absence and presence of Si) on the uptake of N and P, stoichiometric homeostasis of C:N:P, nutritional efficiency, photosynthesis, and dry matter production of maize plants. Suppose the hypotheses of this study are accepted. In that case, it should open new research paths by expanding knowledge on the mechanisms of the mitigating action of Si in plants under stress caused by K deficiency and its relationship with the nutritional efficiency of key nutrients in plant metabolism (C, N, and P). Thus, using Si would become another sustainable strategy for maize cultivation in environments with low K availability with global implications, given the vast area deficient in K in different growing regions.

## Results

### C, N, and P and Si concentrations

**Figure 1:**
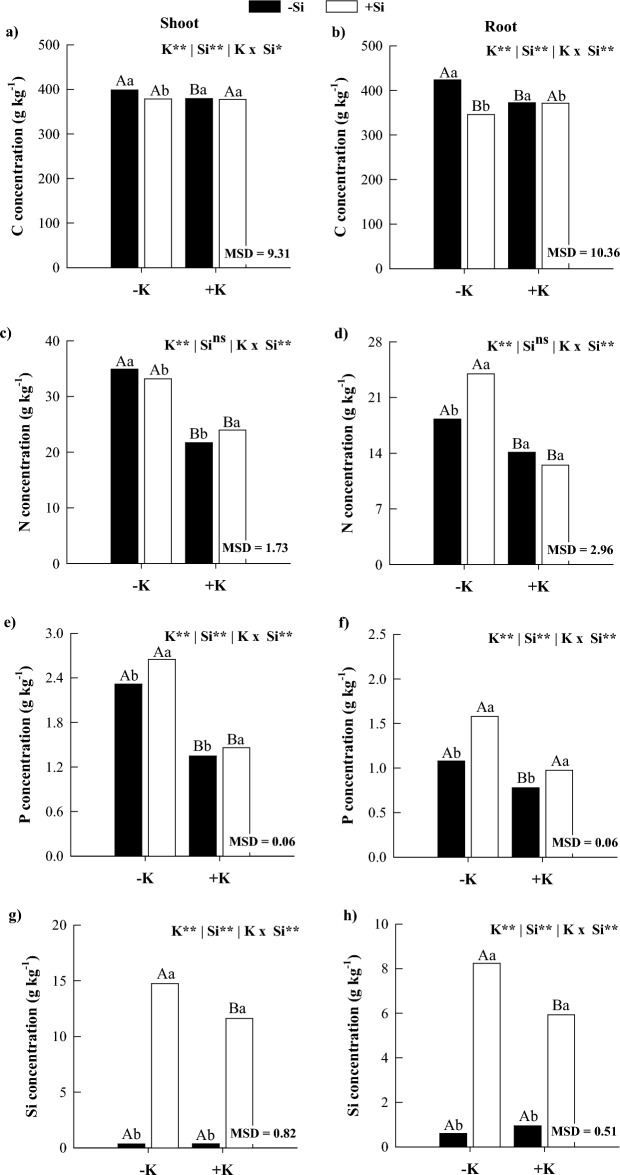
Concentrations of C (**a**,**b**), N (**c**,**d**), P (**e**,**f**), and Si (**g**,**h**) in shoots and roots of maize plants cultivated in the absence (0 mg L^−1^) and presence of Si (56.17 mg L^−1^) under two levels of K (deficiency and sufficiency). Distinct lowercase letters represent a significant difference in Si supply, and distinct capital letters represent a significant difference in K levels at 5% probability by Tukey's test. MSD: minimum significant difference.

In the absence of Si, K deficiency increased C, N, and P concentrations in the shoots and roots of maize plants (Fig. 1) compared with K sufficiency. Silicon supply reduced C and N concentrations in the shoot and the C concentration in the root of K-deficient maize plants. However, it increased P and Si concentrations in the shoot and N, P, and Si concentrations in the root of maize plants (Fig. 1). In plants under K sufficiency, Si supply decreased the C concentration and increased N, P, and Si concentrations in the shoot and P and Si concentrations in the root (Fig. 1).

### C:N:P ratios

**Figure 2:**
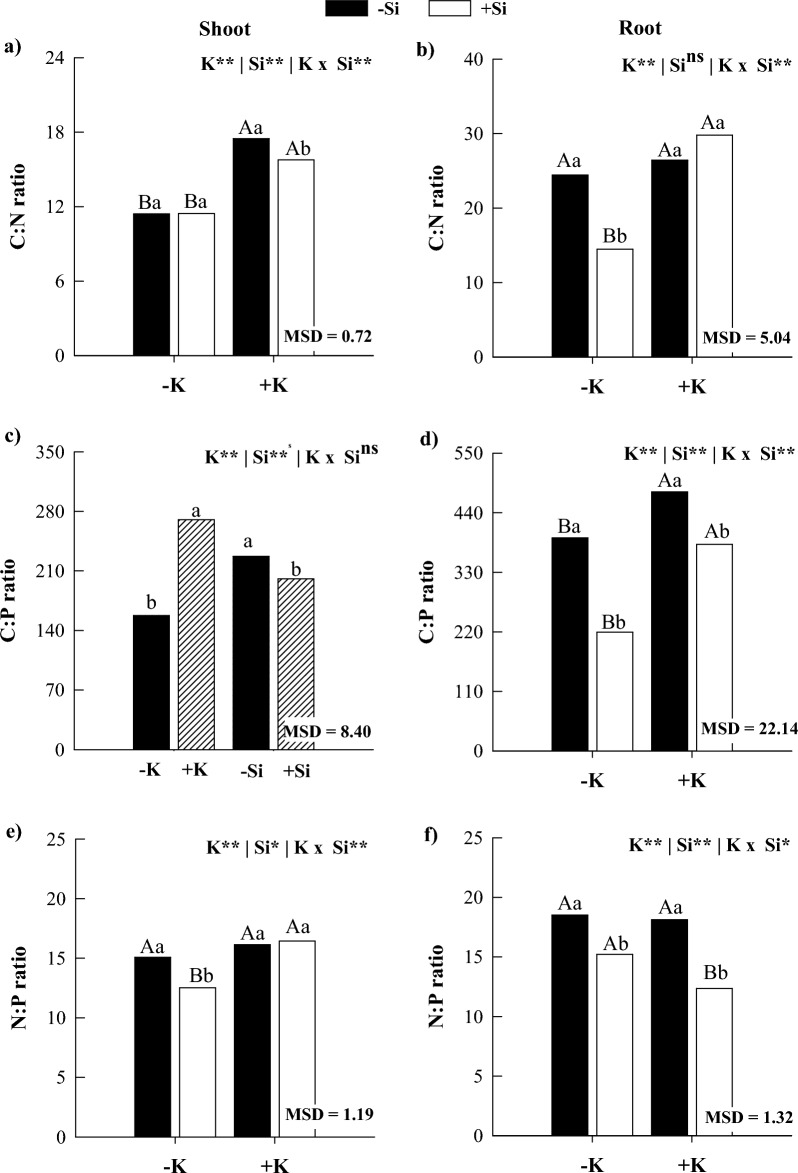
C:N (**a**,**b**), C:P (**c**,**d**), and N:P (**e**,**f**) ratios in shoots and roots of maize plants cultivated in the absence (0 mg L^−1^) and presence of Si (56.17 mg L^−1^) under two levels of K (deficiency and sufficiency). Distinct lowercase letters represent a significant difference in Si supply, and distinct capital letters represent a significant difference in K levels at 5% probability by Tukey’s test. *MSD* minimum significant difference.

**Figure 3:**
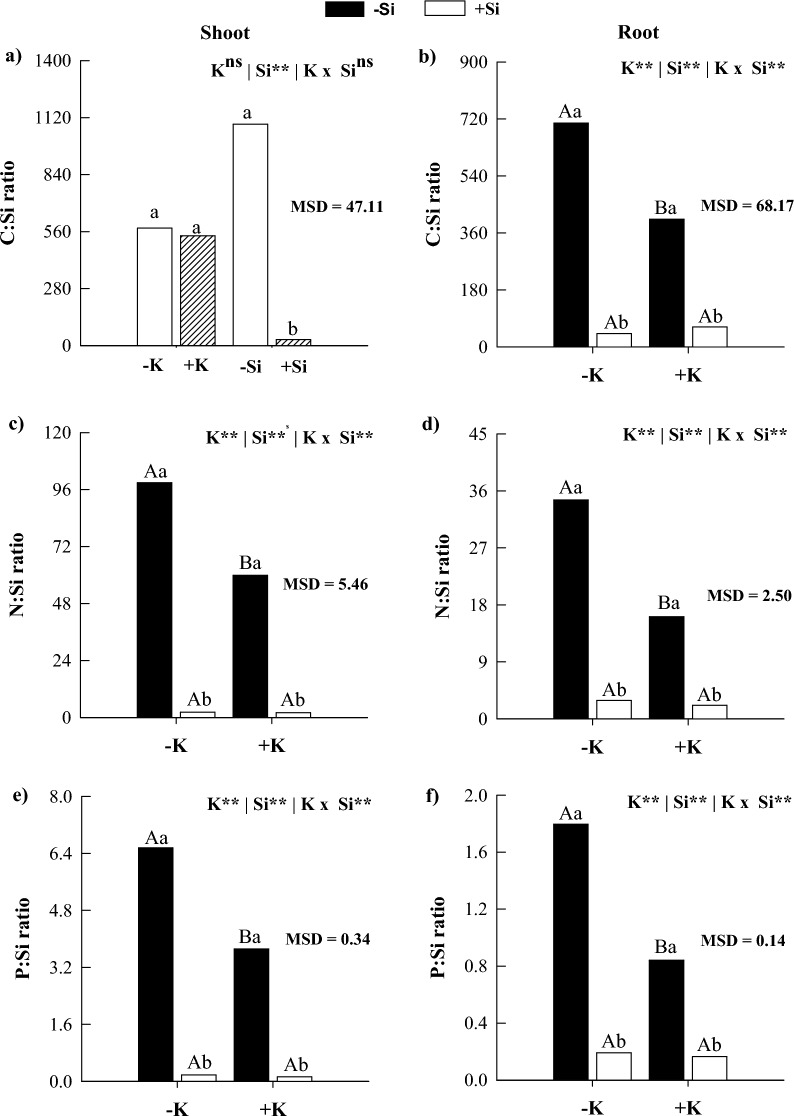
C:Si (**a**,**b**), N:Si (**c**,**d**), and P:Si (**e**,**f**) ratios in shoots and roots of maize plants cultivated in the absence (0 mg L^−1^) and presence of Si (56.17 mg L^−1^) under two levels of K (deficiency and sufficiency). Distinct lowercase letters represent a significant difference in Si supply, and distinct capital letters represent a significant difference in K levels at 5% probability by Tukey’s test. *MSD* minimum significant difference.

In the absence of Si, K deficiency decreased the C:N ratio in the shoot and the C:P ratio in the root while increasing the C:Si, N:Si, and P:Si ratios in the shoots and roots of maize plants compared with K sufficiency (Figs. 2 and 3). The application of Si in K-deficient plants reduced C:N, C:P, N:P, C:Si, N:Si, and P:Si ratios in shoots and roots, except for C:N and C:Si in shoots and C:P in roots (Figs. 2 and 3). In plants under K sufficiency, Si supply decreased C:N, C:P, N:P, C:Si, N:Si, and P:Si ratios in shoots and roots, except for the N:P ratio in the shoot and C:N ratio in the root (Figs. 2 and 3). The results obtained for the C:P and C:Si ratios in the aboveground part responded independently, showing no interaction between the factors. A significant reduction in the C:P ratio was observed under K deficiency conditions, which was mitigated by silicon application (Fig. 2c). On the other hand, the C:Si ratio showed a substantial increase under K deficiency. Meanwhile, silicon application resulted in an additional increase in the stoichiometric ratio (Fig. 3a).

### C, N, and P and Si content

**Figure 4:**
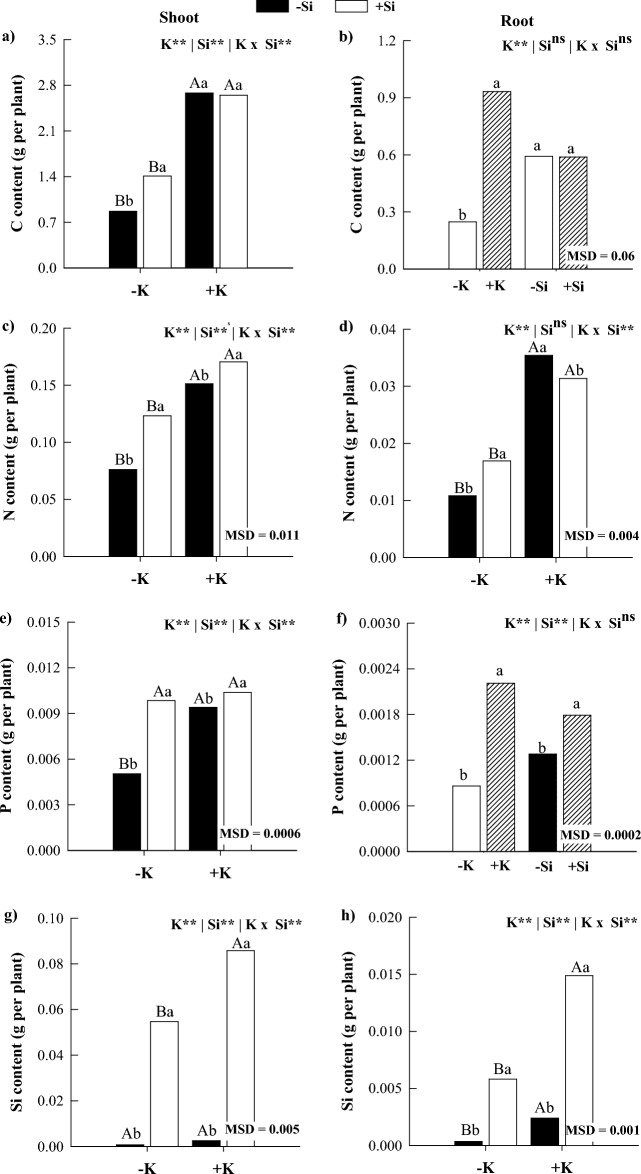
Contents of C (**a**,**b**), N (**c**,**d**), P (**e**,**f**), and Si (**g**,**h**) in shoots and roots of maize plants cultivated in the absence (0 mg L^−1^) and presence of Si (56.17 mg L^−1^) under two levels of K (deficiency and sufficiency). Distinct lowercase letters represent a significant difference in Si supply, and distinct capital letters represent a significant difference in K levels at 5% probability by Tukey’s test. *MSD* minimum significant difference.

In the absence of Si, K deficiency decreased C, N, P, and Si contents in shoots and N and Si contents in roots of maize plants compared to K sufficiency (Fig. 4). In K-deficient plants, Si supply increased C, N, P, and Si contents in shoots and roots of maize plants, except for the C and P content in roots (Fig. 4). In plants under K sufficiency, Si supply increased N, P, and Si contents in shoots and P and Si contents in roots of maize while decreasing the N content in roots (Fig. 4). The accumulation of C and P responded independently, with a reduction in the accumulation of these nutrients in the presence of K deficiency and an increase in P accumulation with Si application (Fig. 4b,f).

### Nutritional efficiency

**Figure 5:**
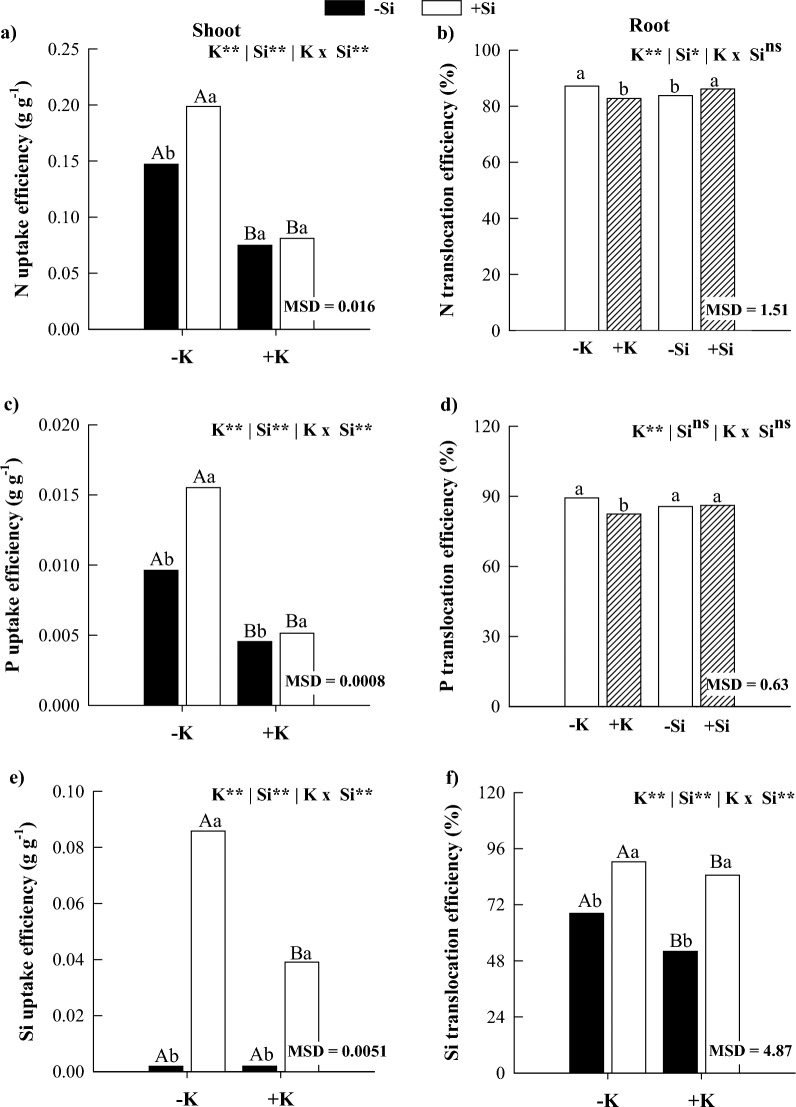
Uptake and translocation efficiency of N (**a**,**b**), P (**c**,**d**), and Si (**e**,**f**) from maize plants cultivated in the absence (0 mg L^−1^) and presence of Si (56.17 mg L^−1^) under two levels of K (disability and sufficiency). Distinct lowercase letters represent a significant difference in Si supply, and distinct capital letters represent a significant difference in K levels at 5% probability by Tukey’s test. *MSD* minimum significant difference.

In the absence of Si, K deficiency increased N and P uptake efficiency and N, P, and Si translocation efficiency compared with K sufficiency (Fig. 5). Silicon supply increased N, P, and Si uptake efficiency in maize plants under K deficiency and sufficiency, except for the N uptake efficiency in plants under K sufficiency (Fig. 5). Furthermore, Si translocation efficiencies increased with the presence of Si in maize plants under K deficiency and sufficiency (Fig. 5). Regarding the translocation efficiency of N and P, there was an isolated response of the factors, with an increase in the translocation efficiency of these nutrients under K deficiency. In addition, when Si was applied, there was an increase in the translocation efficiency of N (Fig. 5b,d).

**Figure 6:**
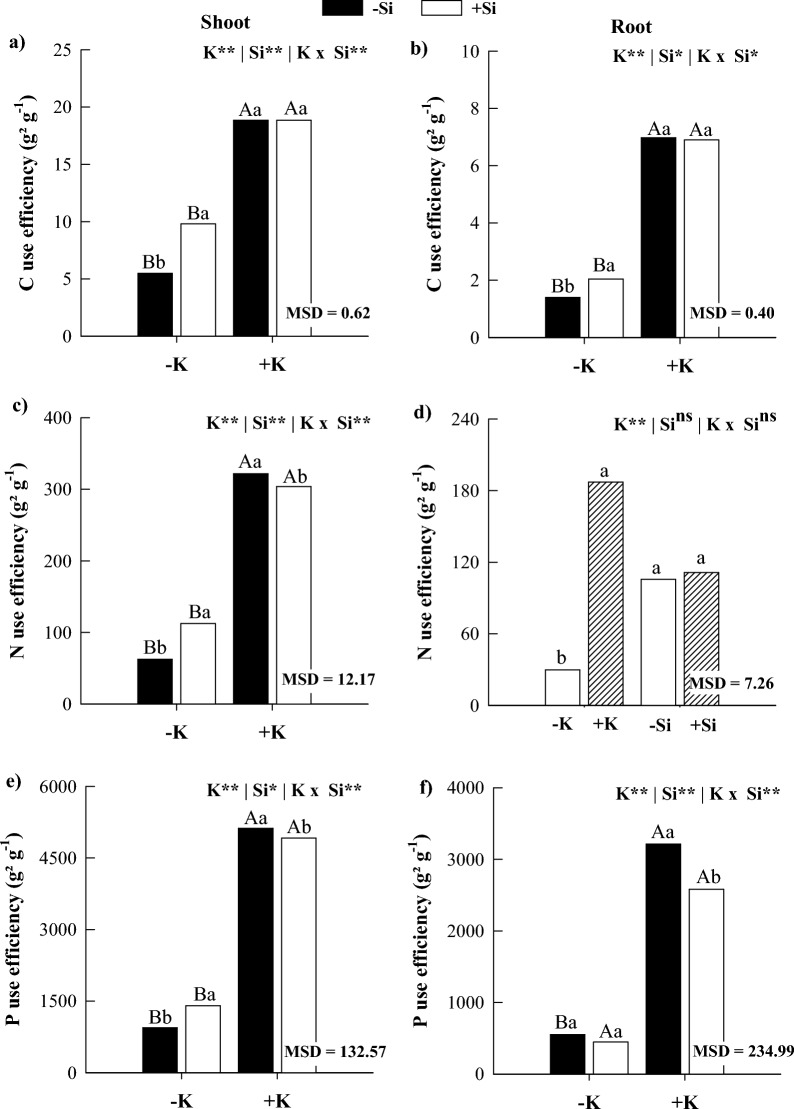
Use efficiency of C (**a**,**b**), N (**c**,**d**), and P (**e**,**f**) in shoots and roots of maize plants cultivated in the absence (0 mg L^−1^) and presence of Si (56.17 mg L^−1^) under two levels of K (deficiency and sufficiency). Distinct lowercase letters represent a significant difference in Si supply, and distinct capital letters represent a significant difference in K levels at 5% probability by Tukey's test. *MSD* minimum significant difference.

In the absence of Si, K deficiency in maize plants decreased the use efficiency of C, N, and P in shoots and C and P in roots compared to K sufficiency (Fig. 6). Silicon supply increased the use efficiency of C, N, and P in shoots and C in roots of K-deficient maize plants. Meanwhile, it decreased the use efficiency of N and P in shoots and of P in roots of plants under K sufficiency, but with an increase in the use efficiency of N in roots organ (Fig. 6). There was no significant interaction response for N use efficiency, with isolated responses of the factors observed. There was a reduction in N use efficiency in the roots under K deficiency, but there was no significant response to Si application (Fig. 6d).

### Net photosynthesis and dry matter production

**Figure 7:**
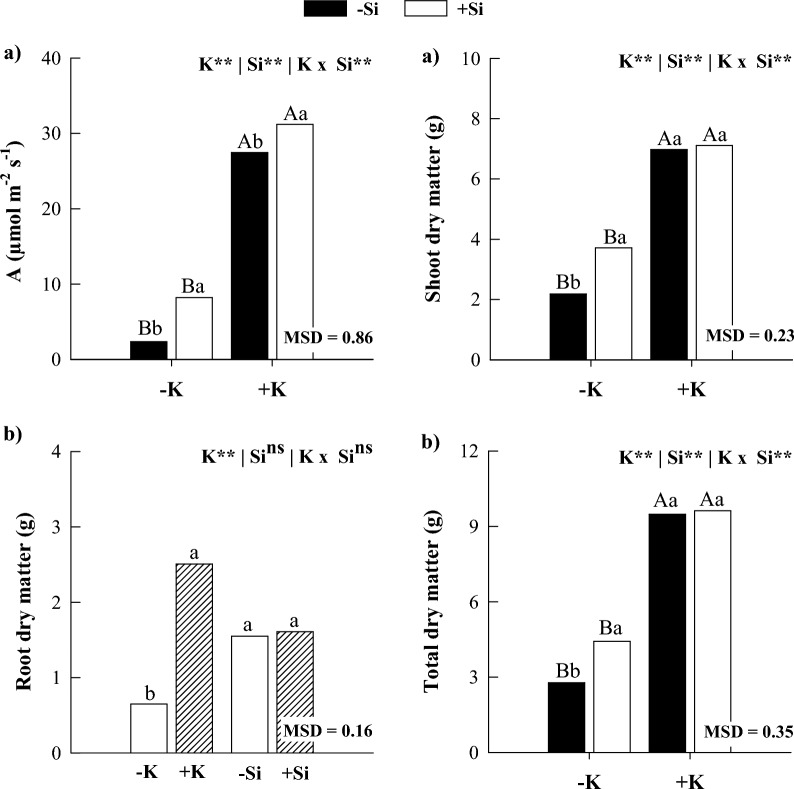
Net photosynthesis (*A*) (**a**) and dry matter production in the shoots (**b**), roots (**c**), and total dry matter production (**d**) of maize plants grown in the absence (0 mg L^−1^) and presence of Si (56.17 mg L^−1^) under two levels of K (deficiency and sufficiency). Distinct lowercase letters represent a significant difference in Si supply, and distinct capital letters represent a significant difference in K levels at 5% probability by Tukey's test. *MSD* minimum significant difference.

In the absence of Si, K deficiency decreased net photosynthesis and shoot, root, and total dry matter production compared with plants under K sufficiency (Fig. 7). Silicon supply in maize plants under K deficiency increased net photosynthesis and shoot and total dry matter (Fig. 7). Silicon supply in maize plants under K sufficiency increased net photosynthesis. However, there was no response in the dry matter production of shoots, roots, and the whole plant (Fig. 7).

### Hierarchical clustering analysis

**Figure 8:**
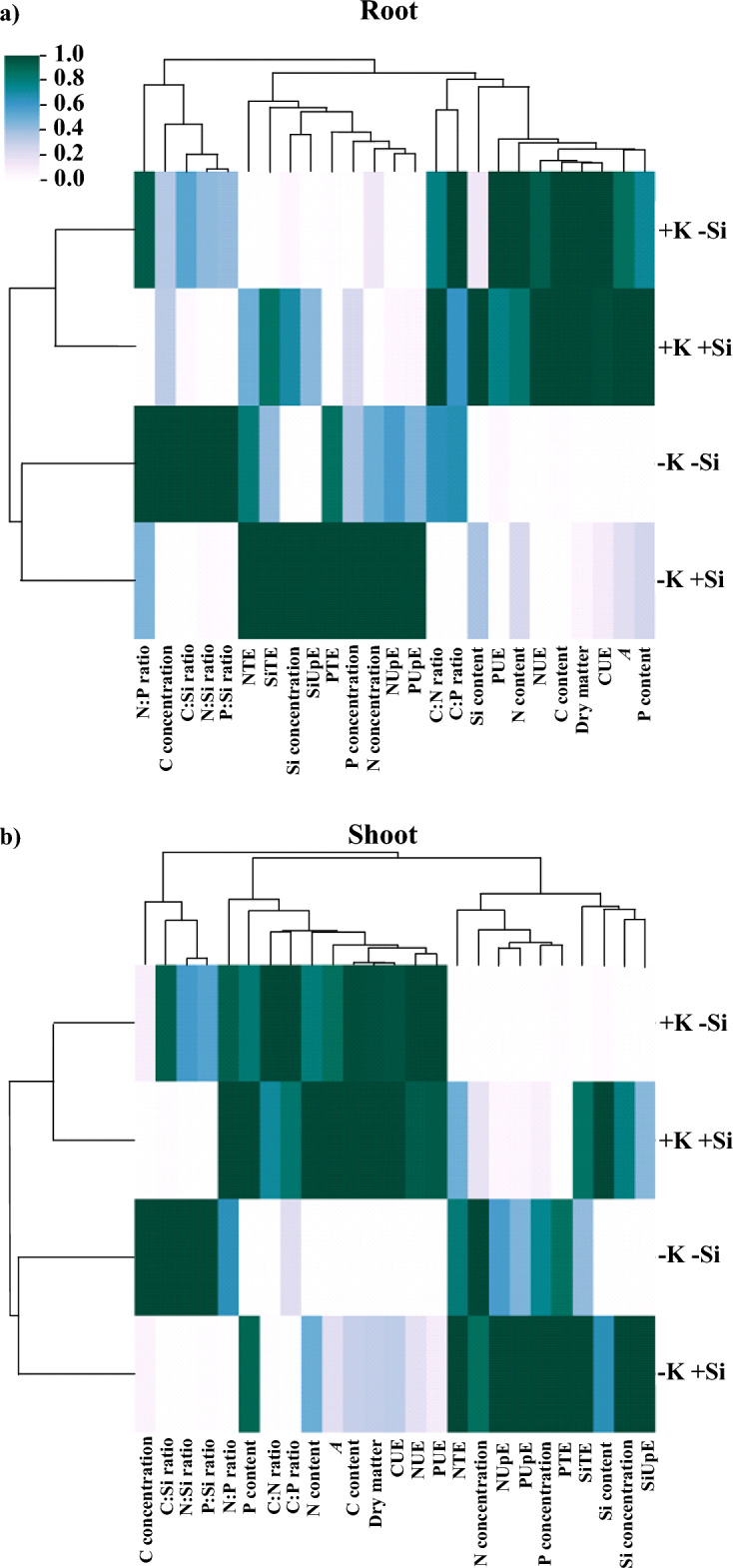
Heat map of the hierarchical grouping of response variables in roots (**a**) and shoots (**b**) of maize plants cultivated in the absence (0 mg L^−1^) and presence of Si (56.17 mg L^−1^) under two levels of K (deficiency and sufficiency). Distinct lowercase letters represent a significant difference in Si supply, and distinct capital letters represent a significant difference in K levels at 5% probability by Tukey’s test. *NTE* N translocation efficiency, *PTE* P translocation efficiency, *SiTE* Si translocation efficiency, *NUpE* N uptake efficiency, *PupE* P uptake efficiency, *SiUpE* Si uptake efficiency, *CUE* C use efficiency, *NUE* N use efficiency, *PUE* P use efficiency, *A* net photosynthesis.

The hierarchical grouping analysis showed greater similarity between treatments with K deficiency (−K−Si and −K + Si) in shoots and roots of maize plants (Fig. 8).

In maize roots, increased C and N contents, C, N, and P use efficiencies, net photosynthesis, and dry matter were associated with K sufficiency, regardless of Si supply (Fig. 8a). Increased C concentrations and C:Si, N:Si, P:Si, and N:P ratios were associated with K deficiency without Si supply. Meanwhile, increased N, P, and Si concentrations, uptake efficiencies, and translocations were associated with K deficiency and Si supply (Fig. 8a).

In the shoot of maize plants, the increase in use efficiencies of C, N, and P, C content, N:P ratio, net photosynthesis, and dry matter were associated with the two conditions of K sufficiency (presence and absence of Si) (Fig. 8b). Moreover, the increase in N, P, and Si contents and N:P ratio were associated with the condition of K sufficiency with Si supply. Meanwhile, the increase in C and N concentrations and C:Si, N:Si, and P:Si ratios were associated with K deficiency without Si supply (Fig. 8b). Finally, the increase in P and Si concentrations, N, P, and Si uptake efficiencies, and N, P, and Si translocation efficiencies were associated with K deficiency with Si supply (Fig. 8b).

### Principal component analysis

**Figure 9:**
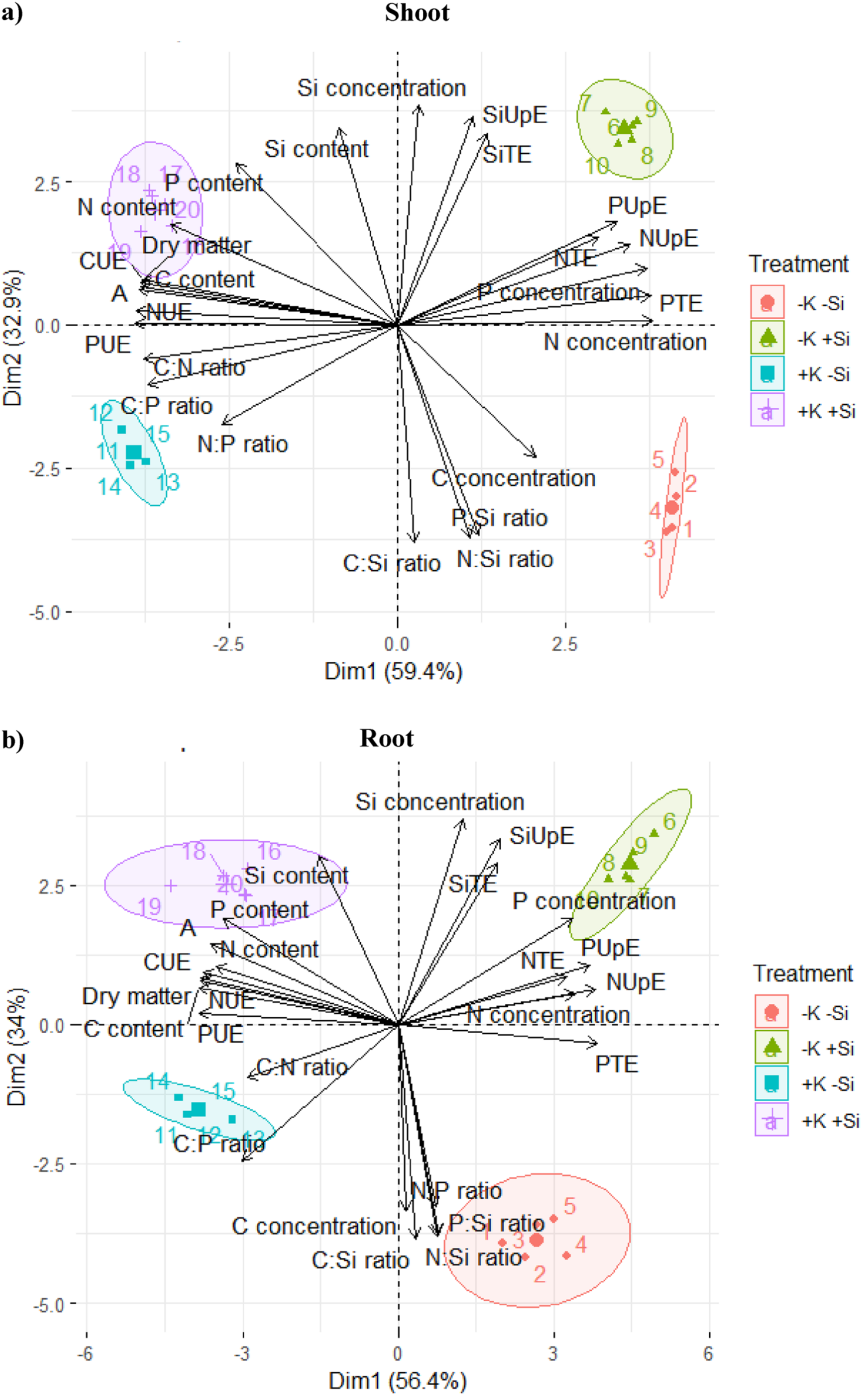
Principal component analysis of the response variables in shoots (**a**) and roots (**b**) of maize plants cultivated in the absence (0 mg L^−1^) and presence of Si (56.17 mg L^−1^) under two levels of K (disability and sufficiency). Distinct lowercase letters represent a significant difference in Si supply, and distinct capital letters represent a significant difference in K levels at 5% probability by Tukey’s test. *NTE* N translocation efficiency, *PTE* P translocation efficiency, *SiTE* Si translocation efficiency, *NUpE* N uptake efficiency, *PupE* P uptake efficiency, *SiUpE* Si uptake efficiency, *CUE* C use efficiency, *NUE* N use efficiency, *PUE* P use efficiency, *A* net photosynthesis.

Principal component analysis (PCA) in the shoot of maize plants explained 92% of the total variance, with PC1 explaining 59% and PC2 explaining 33% (Fig. 9a). For PCA in the shoot, the variables of dry matter, net photosynthesis, N and P concentrations, C:N ratio, C, N, and P use efficiencies, C and N contents, and N and P uptake efficiencies contributed to explaining the variance in PC1. Carbon and Si concentrations, C:Si, N:Si, and P:Si ratios, Si uptake efficiency, and N, P, and Si translocation efficiencies contributed to explaining the variance in PC2. In turn, the P content and C:P ratio contributed to explaining the variance in PC1 and PC2 (Fig. 9a). Principal Component Analysis indicated an association between an increased C concentration and C:Si, N:Si, and P:Si ratios in the treatment under K deficiency in the absence of Si. Meanwhile, the increases in N, P, and Si concentrations, N, P, and Si uptake efficiencies, and N, P, and Si translocation efficiencies were associated with the treatment under K deficiency in the presence of Si (Fig. 9a). The variables of C, N, P, and Si content, C, N, and P use efficiencies, net photosynthesis, and dry matter were associated with the treatment under K sufficiency in the presence of Si. In contrast, C:N, C:P, and N:P ratios were associated with the treatment under K sufficiency in the absence of Si (Fig. 9a).

In the principal component analysis (PCA), roots of maize plants explained 90% of the total variance, with PC1 explaining 56% and PC2 explaining 34% (Fig. 9b). For PCA in the root, variables dry matter, net photosynthesis, N and P concentrations, C:N and C:P ratios, C content, C, N, and P use efficiencies, N uptake, and P and Si translocation efficiencies contributed to explaining the variance in PC1. Carbon and Si concentrations, C:Si, N:Si, and P:Si ratios, P and Si content, and Si uptake efficiency contributed to explaining the variance in PC2. In turn, the N, N:P ratio, P uptake efficiency, and N translocation efficiency contributed to explaining the variance in PC1 and PC2 (Fig. 9b). The PCA showed that the variables C concentration and C:Si, N:Si, P:Si, and N:P ratios were associated with K deficiency in the absence of Si. Meanwhile, the increase in N, P, and Si, N, P, and Si uptake efficiencies, and N and Si translocation efficiencies were associated with the K-deficient treatment in the presence of Si (Fig. 9b). Furthermore, the increase in C:N and C:P ratios were associated with K sufficiency in the absence of Si. In contrast, the increase in C, N, P, and Si contents, C, N, and P use efficiencies, net photosynthesis, and dry matter were related to K sufficiency in the presence of Si (Fig. 9b).

## Discussion

### New aspects on the effects of K deficiency in maize plants without Si application

The damage caused by K deficiency in maize growth is commonly explained as biochemical and physiological damage to the plants^34,37–39^. However, these authors do not mention damage to the C:N:P homeostasis. Our results reveal that K deficiency changes the balance of C:N, C:P, N:P, C:Si, N:P, and P:Si ratios in shoots and of C:P, C:Si, N:Si, and P:Si ratios in roots of maize plants (Figs. 2 and 3). The impacts of K deficiency significantly changed the homeostasis of C, N, and P, decreasing these nutrients’ use efficiency in shoots and roots, reducing the ability of plants to convert accumulated nutrients into dry matter (Fig. 6), and decreasing the dry matter (Fig. 7). Other studies conducted on grasses have also revealed that the homeostatic balance of C, N, and P is affected under stress conditions^19,21,40,41^. A recent study on bean plants under K deficiency conditions showed negative impacts on the C, N, and P balance^33^. This study’s findings align with the existing literature, highlighting that stressful conditions can disrupt plants’ C, N, and P balance, particularly under K deficiency.

Many studies on different species indicate that K deficiency decreases N and P uptake^8–12^, which was also observed in maize plants, as it decreased nutrient accumulation. In other words, the amount of nutrients accumulated in the plant (Fig. 3). Potassium-deficient plants present a decreased transport of carbohydrates to the roots, which could be metabolized to increase energy production in the membrane transporters for N and P^42^. Considering the low content of N and P in the plants aggravated by K deficiency, the functions of N and P in the plants are compromised. Thus, the low use efficiency of N and P, which are indispensable in the photosynthetic process of plants^7,43^, explains the decrease in net photosynthesis in maize plants under K deficiency. Therefore, we can accept the first hypothesis, as it was evidenced in maize that K deficiency, in addition to the damage already discussed in the literature^27–32,34^, also disturbs the stoichiometric homeostasis of macronutrients to the extent of compromising the plan’s C, N, and P nutritional efficiency and its growth. A study on maize plants under K deficiency also provides evidence of the negative impact on the photosynthetic process, supporting our findings^34^.

Furthermore, it is worth pointing out that the results of hierarchical grouping analysis complement that K deficiency is associated with the decrease in dry matter, C, N, P, and Si accumulation, and C, N, and P use efficiency, also increasing the C concentration and N:P, C:Si, N:Si, and P:Si ratios (Fig. 8). This study reinforces the weight of changing C:N:P:Si stoichiometry in the nutritional losses of maize plants under K deficiency.

Thus, our discovery indicates that this disorder caused by K deficiency is serious in plant metabolism. Moreover, it is a reality in many crops worldwide due to the use of underdoses of this nutrient, as the international costs of this fertilizer have been increasing, which worsened after the recent war in Ukraine. In addition, there is a lack of financial resources in many countries, especially underdeveloped nations, to purchase this fertilizer. Therefore, there is a need to urgently reduce the damage caused by this nutritional stress in crops. In this context, using Si can be a sustainable alternative, as it does not damage the environment while also potentially increasing the growth of these crops, mitigating this problem. However, these Si benefits need to be proven through research.

### New impacts of Si in mitigating K deficiency in maize plants

The latest evidence has demonstrated the role played by Si in mitigating K deficiency in maize plants by reducing oxidative stress^34^ favoring gene expression^44^ and mitigating nutritional stress. However, a question has arisen regarding whether the benefit of Si in K-deficient plants may be related to the modification of the C:N:P homeostatic balance. Our results revealed that under K deficiency, Si application could decrease stoichiometric ratios related to C, N, and P in maize plants, modifying these nutrients’ homeostasis.

Thus, Si supply in K-deficient plants reduced the N:P ratio in shoots of maize plants (Fig. 2e,f) from 15 to 12. The adequate range of this ratio is from 10 to 20^45^. However, other authors indicate that if this ratio is greater than 14, it may suggest P limitations^46^. Therefore, using Si may decrease the risk of P limitation in the plant. In this context, Si could decrease the N:P ratio since there is a positive interaction between P and Si, improving the P status in plants. The improvement of P status promoted by Si also decreased the C:P ratio in shoots and roots of maize plants cultivated under K deficiency (Fig. 2c,d). Silicon promotes a better P status in K-deficient plants by increasing the efficiency of P uptake in maize plants, as there are reports that this beneficial element increases the activity of P transporters in plants (TaPHT1;1 and TaPHT1;2)^36^. A study on scarlet eggplant plants also found that silicon reduced the C:N ratio under stress conditions caused by phosphorus toxicity^47^. Similarly, in sugarcane plants experiencing water deficit, applying Si also reduced the C:N ratio^19,20,41^.

Furthermore, Si supply in K-deficient plants decreased the C:N ratio, especially in the plant root, since the efficiency of N uptake improved (Fig. 5a). Therefore, the decrease in the C:N ratio in plants fertilized with Si is related to the improvement in N uptake efficiency (Fig. 5a). The greater uptake of N probably occurred due to the effect of Si on the gene expression of nitrate transporters (BnaNTR2.1)^36^. The C:N ratio variation in plants may indicate a limitation of N, consequently hampering plant growth^48^. Despite the reduction in the stoichiometric C:N ratio in the roots, there was no significant response in the C:N ratio in the shoot of the plants (Fig. 3a). This may be related to the reduction in C and N concentrations in the aboveground part, maintaining the same stoichiometric ratios when compared to the absence of Si. Other studies have also reported a reduction in nitrogen concentration in grasses^49^ and rice^50^. This phenomenon may occur due to the increase in dry mass in the aboveground part of the plants (Fig. 7d). Despite the enhanced efficiency of N uptake, translocation, and content in the aboveground part induced by Si, N dilution in plant tissues can still occur.

Moreover, it is worth noting that the effect of Si on the reduction of C:N and C:P ratios in K-deficient plants, as mentioned above, is not only provided by its benefit in increasing the uptake of N and P. It is also related to the decrease in the C concentration that occurred in the leaves and roots of plants (Fig. 1a,b). This effect of Si on the reduction of C in the plant draws attention since this fact is attributed to the biological role played by Si in partially replacing C in cell wall formation^51^, which reduces the demand for C in plant tissues. The exchange of C for Si in the cell wall occurs due to the lower energy cost of incorporating Si since the investment of 1 g of glucose can incorporate 0.86 g of carbohydrates or 12.67 g of SiO_2_^51^. That is why there is a tendency for greater Si uptake by plants under stress conditions, such as K deficiency compared to K sufficiency (Fig. 4g,h). Thus, it indicates that the accumulation of more Si to prevent damage from stress is a plant defense mechanism^52^. This result is surprising because it contradicts the reduction in Si uptake observed under water deficit conditions^20,53^, indicating that under K deficiency, Si supply is not limited, allowing for greater utilization of this beneficial element.

The alleviating role played by Si in maize plants under K deficiency was explained mainly by the increase not only in the accumulation of N and P but also by the improvement in these nutrients’ uptake and translocation efficiency. Thus, it results in the increase of their concentration, as evidenced by the PCA (Fig. 9) and plant dry matter (Fig. 7d). In this scenario, Si contributes to improving the status of N and P in maize plants, which are impaired by K deficiency, reflecting on these nutrients’ homeostatic balance (Figs. 2 and 3).

Thus, the improvement in the previously discussed stoichiometric ratios promoted by Si in K-deficient plants was sufficient to improve C, N, and P use efficiency (Fig. 6) through an increase in net photosynthesis (Fig. 7a), as N and P are vital for different physiological processes^7^. In addition to the effect of Si in reducing energy expenditure to comprise cell wall components with Si instead of C, which was previously shown by Raven (1983), this fact explains the benefit of Si in increasing dry matter production, especially in the shoot of maize. Therefore, the second hypothesis can be accepted, indicating that Si can modify the C:N:P stoichiometry enough to improve the nutritional efficiency and photosynthetic rate of maize, consequently increasing dry matter production. Furthermore, it is worth noting that Si was more important for increasing shoot dry matter than root dry matter in maize plants under K deficiency. It may occur because, in plants accumulating Si, such as maize, most of the Si is absorbed and transported to shoots due to the presence of efficient Si transporters in this species^54,55^. Therefore, the benefits of Si in increasing biomass predominate in leaf organs compared to the root, which is also evidenced in other species^56–58^. In non-silicon-accumulating plants, such as beans, the results are different. These plants lack efficient transporters, resulting in an increase in root dry mass and no response in the aboveground part^33^. This phenomenon may contribute to the lack of response of Si in increasing P use efficiency in the roots and P translocation, despite the increase in P concentration and content in the roots and the enhancement of P uptake efficiency.

Finally, the hierarchical cluster analysis also visualized the nutritional improvement of Si, which resulted in the mitigation of the nutritional stress in maize plants. It reinforced that Si effectively improved the efficiency of N and P uptake by increasing the concentration of these nutrients in shoots and roots. Meanwhile, it reduced the C concentration and C:Si, N:Si, P:Si, C:P, and N:P ratios of K-deficient maize plants (Fig. 8). This study should open new research paths by expanding knowledge on the mechanisms of Si that mitigate K-deficiency stress, consequently providing a new sustainable strategy for maize cultivation in environments with low K availability.

Another aspect worth mentioning is the fact that our results also showed that the Si supply in maize plants without stress (under K sufficiency), although changing the C:N:P stoichiometry (Figs. 2 and 3), was not enough to increase the use efficiency of these nutrients (Fig. 6) and dry matter production (Fig. 7a–c). It reinforces the fact that the benefits of Si predominate in plants under stress compared to those without stress since this element is not considered a plant nutrient^36^. Thus, we do not recommend Si in fertile areas cultivated with maize under adequate nutritional status because the plant metabolism is optimized, and it is not possible to improve the crop’s nutritional efficiency.

## Conclusions

This study demonstrates that potassium (K) deficiency in maize plants not only causes biochemical and physiological damage but also disrupts the balance of carbon (C), nitrogen (N), phosphorus (P), and silicon (Si) ratios in both shoots and roots. This imbalance affects nutrient use efficiency and hampers the conversion of accumulated nutrients into dry matter, resulting in reduced plant growth. Furthermore, K deficiency leads to decreased uptake and accumulation of nitrogen and phosphorus, limiting their functions in the photosynthetic process.

The results also highlight the mitigating role of silicon (Si) in K-deficient maize plants. Silicon application reduces stoichiometric ratios related to C, N, and P, thereby improving the homeostasis of these nutrients. Silicon supplementation decreases the N:P ratio, suggesting a reduced phosphorus limitation risk. Moreover, Si increases the N uptake efficiency and decreases the C:N ratio, improving nitrogen uptake efficiency. Furthermore, Si contributes to reducing the C concentration by partially replacing carbon in cell wall formation, thereby reducing the demand for carbon in plant tissues.

The beneficial effects of Si on nutrient uptake and translocation, and the improvement in nutritional efficiency and photosynthetic rate, result in increased dry matter production, particularly in the shoot of maize plants. Silicon is more effective in increasing shoot dry matter compared to root dry matter in K-deficient maize plants, likely due to the efficient transport of Si to shoots in Si-accumulating species, such as maize. These findings suggest that Si could represent a sustainable alternative to mitigate the detrimental effects of K deficiency in maize crops.

However, it should be noted that Si supplementation did not significantly improve nutrient use efficiency or dry matter production in maize plants under K sufficiency. Therefore, Si application is not recommended in fertile areas with maize crops with adequate nutritional status.

In conclusion, this study provides new insights into the effects of K deficiency and the potential of Si supplementation in maize plants. The findings emphasize the importance of addressing K deficiency-related issues in crop production, particularly in regions where K availability is limited due to cost and resource constraints. Silicon supplementation could offer a sustainable solution to mitigate the negative impacts of K deficiency and improve maize cultivation in low-K environments. Further research must deepen our understanding of the mechanisms underlying Si’s beneficial effects in alleviating K deficiency stress in plants.

## Methods

### Experimental conditions

The study was carried out with maize plants under controlled greenhouse conditions at the School of Agricultural and Veterinary Sciences (FCA) of the São Paulo State University Júlio Mesquita Filho (UNESP), in the municipality of Jaboticabal, São Paulo, Brazil. Climatic conditions were monitored during the experiment using a digital thermo-hygrometer, through which the relative air humidity (32.8 ± 8%), minimum temperature (19.5 ± 5 °C), and maximum temperature (38.6 ± 7 °C) were recorded.

### Experimental design and setup

The experiment included a randomized block design in a 2 × 2 factorial scheme. It comprised two K concentrations (deficiency with 7.82 mg L^−1^ and sufficiency with 234.59 mg L^−1^) and two Si concentrations (absence with 0.0 mg L^−1^ and presence with 56.17 mg L^−1^) with five replicates. The silicon dose used in this study was based on previous research in maize cultivation^59–62^ and focused on maximum crop response.

Sowing was conducted in a tray filled with washed sand (water, 1% HCl solution, and deionized water) using maize seeds of the cultivar 2B633PW (Dow AgroSciences). After seed emergence, a seedling was transplanted to each experimental plot, comprising a 10-L polypropylene tray (0.44 × 0.19 × 0.14 m) filled with washed sand. After transplanting, nutrient solution^63^ was applied. Potassium levels (deficiency and sufficiency) were adjusted using a solution concentration of 10% in the first week, 25% in the second week, and 50% from the third week until the end of the experiment. The solution’s pH was adjusted to a range of 5.5–6.5 with standard NaOH (1 mM) and HCl (1 mM) solutions. We used a dropper to add drops of NaOH or HCl solutions until the desired pH range was reached without exceeding 1 mL of the NaOH or HCl standard solutions. At weekly intervals, the substrates were drained to eliminate excess salt using 700 mL of deionized water.

Potassium was supplied via nutrient solution in the form of potassium chloride (KCl) using a concentration of 226.77 mg L^−1^ of K. Silicon was also applied via nutrient solution in pre-established treatments using stabilized sodium and potassium silicate with sorbitol (SiNaKE) (113.4 g L^−1^ of Si; 18.9 g L^−1^ of K_2_O; 60.5 g L^−1^ of Na_2_O; and 100 mL L^−1^ of sorbitol), balancing the K (7.82 mg L^−1^) with KCl in treatments with absence of Si.

### Experimental evaluations

#### Biomass production

Biomass production was evaluated 26 days after transplanting the seedlings by collecting the plants and separating them into shoots and roots, as the full establishment of potassium deficiency symptoms in the plants had occurred by that time.. After cutting the plants, the samples were decontaminated in deionized water, neutral detergent solution (0.3%), HCl solution (0.1%), and deionized water. Then, the samples were deposited on Kraft paper and dried in an oven with forced air circulation (65 ± 5 °C) until reaching constant weight. Finally, we determined the dry weight of shoots and roots using a semi-analytical scale.

#### Determination of concentrations of C, N, P, and Si

After drying plant tissue samples, they were ground using a Willey knife mill. Carbon concentration was determined by dichromate oxidation in an acid medium and titration of excess Cr^6+^^64^. In order to determine N, the Kjeldahl method involving wet oxidation was used, while P was determined using the nitric-perchloric digestion method and colorimetry with ammonium metavanadate^65^. Silicon concentration was determined by the alkaline digestion method and colorimetry with ammonium molybdate^66^.

#### Elemental stoichiometry

The stoichiometric ratios of the nutrients were estimated from the ratios of the concentrations in the shoots and roots of maize plants, through which the ratios of C:N, C:P, C:Si, N:P, N:Si, and P:Si were established.

#### Elemental content

Elemental contents (C, N, P, and Si) were estimated from the product of the multiplication of the concentration and dry matter in the shoots and roots of maize plants (Eq. 1).

1 $$Content \left(g\,per\,plant\right)=\left[chemical\,element \left({g\,kg}^{-1}\right)\right] x \frac{dry\,matter\,(g)}{1000}$$

#### Nutritional efficiency

The uptake efficiency of N, P, and Si was estimated from the ratio of the total content of the element in the plant and the root dry matter (Eq. 2)^67^.

2 $$Uptake\,efficiency \left({g\,g}^{-1}\right)=\frac{(Shoot\,content+Root\,content) }{Root\,dry\,matter}$$

The translocation efficiency of N, P, and Si was calculated by multiplying the ratio of nutrient content in the shoot and the total nutrient content in the plant by 100 (Eq. 3)^68^.

3 $$Translocation\,efficiency \left(\%\right)= \frac{Shoot content}{(Shoot\,content+Root\,content)}\times 100$$

Nutrient use efficiency (C, N, and P) was calculated from the squared dry matter and nutrient content for plant shoots and roots^69^ (Eq. 4).

4 $$ Use\,efficiency \left( {g\,g^{2} } \right) = \frac{{Dry\,matter^{2} }}{Content} $$

#### Net photosynthesis

Net photosynthesis was evaluated 25 days after seedling transplanting using an Infrared Gas Analyzer (IRGA; LcPro-SD, ADC BioScientific Ltd., Hoddesdon, United Kingdom) in the upper abaxial third of the fourth leaf at 9:00–11:00 h. The photosynthetic photon flux density used in the IRGA camera was 1200 μmol m^−2^ s^−1^.

### Statistical analysis

#### Preposition tests

The data were submitted to the Shapiro–Wilk normality test (p > 0.05)^70^ and Levene's homogeneity test (p > 0.05)^71^ using the Python programming language (version 3.9.7; Python Software Foundation).

#### Analysis of variance and mean test

Analysis of variance was performed (p < 0.05), and when significant, data were submitted to Tukey’s test (p < 0.05) using the Python programming language (version 3.9.7; Python Software Foundation).

#### Principal component analysis and hierarchical grouping

In order to investigate the interrelationships between variables and explain their variance in terms of inherent dimensions (components), principal component analysis was performed, which was calculated from the covariance matrix. Furthermore, a hierarchical grouping analysis of the data was carried out based on Euclidean distance, while cluster analysis was carried out using the single linkage method. The response variables were grouped for cluster analysis and principal component analysis using the variables of plant shoot and root. Meanwhile, the variables of net photosynthesis and efficiency of uptake and translocation were used in both groupings.

### Research involving plants

The experimental research was carried out following the relevant institutional, national, and international guidelines and legislation, and still, it does not involve an endangered species.

## Data Availability

The datasets generated and/or analyzed during the current study are available from the corresponding author upon reasonable request.
